# In Vitro Comparison of Fracture Resistance of Severely Damaged Primary Anterior Teeth Restored with Different Post and Core Systems

**DOI:** 10.1155/2023/2895892

**Published:** 2023-04-28

**Authors:** Mandana Alamdari Mahd, Payvand Moeiny, Haleh Heshmat, Nahid Askarizadeh

**Affiliations:** ^1^Department of Pediatrics, School of Dentistry, Islamic Azad University of Tehran, Tehran 19395/1495, Iran; ^2^Department of Restorative Dentistry, School of Dentistry, Islamic Azad University of Tehran, Tehran 19395/1495, Iran

## Abstract

**Objectives:**

This study aimed to compare the fracture resistance (FR) of severely damaged primary anterior teeth restored with five different post and core systems.

**Materials and Methods:**

This in vitro, experimental study evaluated 60 extracted primary maxillary central incisors. The teeth were horizontally sectioned at 1 mm above their cementoenamel junction (CEJ), underwent pulpectomy, and their root canals were filled with Metapex paste. After post space preparation and sealing of root fillings with light-cure glass ionomer (1 mm thickness), the teeth were randomly assigned to five groups (*n* = 12) of (1) glass fiber post and everX composite (reinforced with short fibers), (2) glass fiber post and bulk-fill composite, (3) everX composite post and core, (4) bulk-fill composite post and core, and (5) Filtek conventional composite post and core. The teeth underwent 5,000 thermal cycles between 5°C and 55°C, and their FR was measured in a universal testing machine (0.5 mm/min, 148°). The mode of failure was also determined. Data were analyzed by ANOVA and Tukey's test at 0.05 level of significance.

**Results:**

The FR was the highest in fiber post and everX composite, and the lowest in Z250 conventional composite post and core group (*P* < 0.001). The FR of fiber post and everX composite group was significantly higher than that of everX composite post and core (*P* = 0.04), bulk-fill composite post and core (*P* = 0.001), and Z250 composite post and core (*P* < 0.001) groups. The frequency of repairable fractures was the highest in glass fiber post plus everX composite (91.66%) and the lowest in Filtek conventional post and core group (66.66%) (*P* > 0.05).

**Conclusion:**

Within the limitations of this in vitro study, the results showed that restoration of severely damaged primary maxillary central incisors with glass fiber post and everX composite reinforced with short fibers enhanced their FR, and increased the chance of reparability in case of restoration fracture. This technique may be recommended for the restoration of primary anterior teeth since it is simple and saves time.

## 1. Introduction

Extensive restorations of primary anterior teeth are always challenging in pediatric dentistry. Restoration of severely damaged primary anterior teeth is often difficult due to small size of the crown, relatively large pulp chamber, and children's young age and poor cooperation. Such restorations often have low fracture resistance (FR) and break due to inadequate sound residual tooth structure [1]. Thus, many dental clinicians prefer extraction rather than restoration of such teeth. However, many parents insist on restoration, rather than extraction [2]. Also, extraction of anterior teeth adversely affects the child's appearance and smile esthetics, decreases the efficiency of mastication, leads to vertical height loss, and development of parafunctional habits such as tongue thrusting, speech problems, and space loss, and also has adverse effects on personality and behavioral development of children [3].

Thus, endodontic treatment and application of techniques to provide retention are imperative prior to placement of crown or direct restorations. The available treatment options for restoration of such teeth include direct restorations with retention through dentin bonds from the root canal or dentin pins, or indirect restorations by using prefabricated crowns and application of composite resins, which may also require metal or fiber posts [4]. Metal posts, biologic posts, omega-shaped stainless steel orthodontic wires, polyethylene fiber posts, and glass fiber posts are commonly used to provide retention in primary teeth [5]. Recently, some new types of posts were fabricated from short fiber-reinforced flowable composite resin (SFRC), which was first suggested as a restorative material for dentin replacement [6].

The available literature is controversial regarding the effect of different post and core systems for restoration of severely damaged teeth on their FR. In vitro studies by Lassila et al. [7] and Mojarad and Selahbarzin [8] showed that type of post and core had a significant effect on FR of restored teeth. However, Mosharrafian et al. [9] and Seraj et al. [1] demonstrated that restorative technique had no significant effect on FR. On the other hand, Garoushi et al. [10, 11] and Bijelic et al. [12] indicated that restoration of permanent anterior teeth with SFRC provided acceptable FR against high loads.

Considering the gap of information regarding the use of SFRC in primary teeth and controversial results about the efficacy of different post and core systems for restoration of severely damaged anterior teeth, this study aimed to compare the FR of severely damaged primary anterior teeth restored with five different post and core systems. The null hypothesis was that no significant difference would be found in FR of severely damaged primary anterior teeth restored with five different post and core systems.

## 2. Materials and Methods

This in vitro, experimental study was conducted on 60 severely damaged primary maxillary central incisors extracted due to severe caries. The study protocol was approved by the ethics committee of Islamic Azad University, School of Dentistry, Tehran (IR.IAU.DENTAL.REC.1401.015). The sample size was calculated to be 12 in each group according to studies by Mosharrafian et al. [9] and Seraj et al. [1] using one-way ANOVA power analysis of PASS 11 assuming *α* = 0.05, *β* = 0.2, mean standard deviation of 3.7 MPa, and effect size of 0.49.

### 2.1. Eligibility Criteria

Extracted primary maxillary central incisors with almost similar dimensions were selected. Since strip crown size 3 (Kids Crown; Shinhung, Korea) with a cross-sectional diameter of 5 mm was to be used, the selected teeth had ±0.5 mm difference in diameter at 1 mm from their cementoenamel junction (CEJ) as measured by a gauge caliper (Aesculap, Germany). Also, the cervical third of the crown had to be present, two-thirds of the root length had to be intact, and the teeth had no history of pulp therapy. Moreover, the teeth had no root crack or fracture when inspected under a scanning electron microscope (Olympus CX31P microscope, Pars Teb Co., Iran) and had been extracted within the past 6 months.

### 2.2. Tooth Preparation

The teeth were rinsed with saline (Shiraz Serum, Iran), disinfected in 0.5% thymol (Sigma Aldrich, Iran) for 1 week, and stored in distilled water at 4°C until use [9]. Debris was removed by a #15 surgical scalpel blade (ATP, Trinon Co., Germany), and the teeth were cleaned with a disposable prophy brush (Melorin, China) and a low-speed hand-piece underwater coolant. They were then sectioned at 1 mm above their CEJ by a high-speed diamond disc (Crwon Cutter, DFS Diamond Co., Germany). The teeth underwent pulpectomy with hand files (Mani, Japan) to three sizes larger than the initial file, and 1 mm shorter than the apex. The root canals were rinsed with saline, dried with absorbent paper points with 0.4% taper (Data, China), and filled with Metapex paste (Metapex, META, Korea). Next, an excavator was used to remove coronal 4 mm of Metapex and measured with a Williams probe (Fatah teb Co, Iran), and light-cure glass ionomer (Willman & Pein-Glass linear, Germany) was applied over the residual root filling at the post space floor with 1 mm thickness and cured for 40 s using a LED curing unit (Radii, SDI co, Australia) [9]. The teeth were then randomly assigned to five groups (*n* = 12) and each tooth was coded accordingly. The study groups were as follows:

Group 1 Fiber post (Reforpost, Angelus, Brazil) and everX composite resin (everX flow bulk shade, GC, Japan)
Group 2 Fiber post (Reforpost, Angelus, Brazil) and bulk-fill composite resin (Filtek bulk fill A1 shade, 3M ESPE, St. Paul, MN, USA)
Group 3 EverX composite post and core
Group 4 Bulk-fill composite post and core
Group 5 Filtek conventional composite post and core (Filtek Z 250 A1 shade, 3M ESPE, St. Paul, MN, USA)

In all groups, restoration was performed as instructed by the manufacturer. In groups 1 and 2, glass fiber post (Reforpost, Angelus, Brazil) with 1.1 mm diameter was used. The posts were sectioned with a diamond bur and high-speed hand-piece underwater coolant to have 5 mm length and were cleaned with alcohol. The post space was gently air dried with air spray for 1–2 s (to remain slightly moist). Next, Scotchbond Universal (3M ESPE, St. Paul, MN, USA) was rubbed on the residual walls of the post space in two separate layers, and excess adhesive was removed by air spray for 3–5 s. It was then cured for 20 s. Next, Rely-X Luting Plus dual-cure resin cement (Automix, 3M ESPE, USA) was used for the cementation of posts and cured for 20 s as instructed by the manufacturer [1].

Celluloid crowns (Kids crown, Shinhung, Korea) were used for coronal reconstruction such that the enamel was first etched with 35% phosphoric acid (Scotchbond Etchant; 3M ESPE, MN, USA) for 15 s, and after 15 s of rinsing and drying, bonding was performed as described for the posts. It should be noted that the intracanal post length was 3 mm, and crown height was 5 mm in all groups [9].

Groups 1 and 2: After post space preparation, placement of post, and etching and bonding of crown as explained above, in each of the everX Flow bulk shade (GC, Japan) and bulk-fill (Filtek A1 shade, 3M ESPE, St. Paul, MN, USA) groups, a hole was created by a bur (No 835. FG.008, Jota, Switzerland), and composite resin was injected into the celluloid crown and cured from each of the labial, palatal, and incisal surfaces for 40 s.

Groups 3 and 4: After separate etching and bonding of enamel and intracanal dentin as explained above, composite was first cured in the canal for 40 s. Next, composite resin was injected into the celluloid crown and cured from each of the labial, palatal, and incisal surfaces for 40 s [9].

Group 5: After separate etching and bonding of enamel and intracanal dentin as explained above, A1 shade of Filtek Z250 conventional composite (3M ESPE, St. Paul, MN, USA) was incrementally applied in layers with 2 mm thickness (3 mm within the canal and 1 mm above the CEJ in two steps as wedge-shaped layers, and the rest was applied into the celluloid crown). Each layer was cured for 40 s, and layers within the crown were separately cured from the labial, palatal, and incisal surfaces [9].

All restorations were finished by a soft diamond bur (No 862. FG.012, Jota, Switzerland) and polished with aluminum oxide discs (Sof-Lex, Prop On, 3M ESPE, USA). The teeth were then mounted in acrylic resin to 1 mm below their CEJ, and subjected to 5,000 thermal cycles between 5°C and 55°C with a dwell time of 30 s [1].

### 2.3. Measurement of FR

The teeth were transferred to a universal testing machine (Zwick Roell, Ulm, Germany) and subjected to load at a crosshead speed of 0.5 mm/min applied at 148° angle to the midpalatal surface at a 2 mm distance from the incisal edge until fracture [1, 9, 13]. The load at fracture was recorded in Newtons (N) and divided by the load application surface area in square millimeters measured by AutoCAD 2016 software to calculate the FR in megapascals (MPa). The diameter of the canal cross-section and the cross-sectional area of the tooth were separately measured, and drawn in AutoCAD 2016 software. Accordingly, the cross-section of the bonding area (difference between the cross-sectional area of the tooth and canal) was calculated [1, 9].

### 2.4. Assessment of Mode of Failure

The specimens were inspected after fracture and categorized into two groups repairable (fractures above the CEJ) and irreparable (fractures below the CEJ) fractures [9].

### 2.5. Statistical Analysis

Data were analyzed by SPSS version 22. The five groups were compared regarding FR by one-way ANOVA. Pairwise comparisons were performed by Tukey's test. *P* < 0.05 was considered statistically significant.

## 3. Results

### 3.1. FR Results

Table 1 presents the FR of the five groups in Newtons and Table 2 shows the fracture strength of specimens in megapascals. One-way ANOVA showed a significant difference in FR among the five groups (*P* < 0.001). Thus, pairwise comparisons were carried out by Tukey's test (Table 3). The results showed significantly higher FR of teeth restored with fiber post and everX composite than everX composite post and core group (*P* = 0.04), bulk-fill composite post and core group (*P* = 0.001), and Z250 composite post and core group (*P* < 0.001). No other significant differences were found (*P* > 0.05).

### 3.2. Mode of Failure

**Figure 1:**
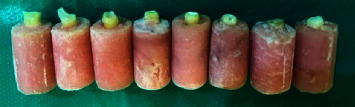
Repairable fractures (above the CEJ).

**Figure 2:**
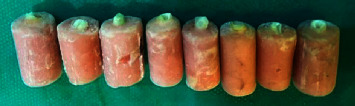
Irreparable fractures (below the CEJ).

Table 4 presents the frequency of repairable and irreparable fractures in the five groups. The frequency of repairable fractures was the highest in glass fiber post plus everX composite (91.66%) and the lowest in Filtek conventional post and core group (66.66%) (*P* > 0.05). Figures 1 and 2 show repairable and irreparable fractures, respectively.

## 4. Discussion

This study compared the FR of severely damaged primary maxillary anterior teeth restored with five different post and core systems. It should be noted that to calculate the flexural strength, the standard dimensions of material are required, which are among the pure properties of a material, and are used for screening purposes, and its assessment was out of the scope of the present study. The objective of the present study was to assess the fracture resistance of tooth, post, and resin complex, and we tried to simulate the clinical conditions in terms of load application (location, and magnitude) as much as possible. The null hypothesis was that no significant difference would be found in FR of severely damaged primary anterior teeth restored with five different post and core systems. The results showed that the FR was the highest in fiber post and everX composite and the lowest in Z250 conventional composite post and core group. The difference in FR was significant among the five groups (*P* < 0.001). Thus, the null hypothesis of the study was rejected. The FR of fiber post and everX composite group was significantly higher than that of everX composite post and core group (*P* = 0.04), bulk fill composite post and core group (*P* = 0.001), and Z250 composite post and core group (*P* < 0.001). The FR of teeth restored with fiber post and everX composite was not significantly different from the FR of teeth restored with fiber post and bulk-fill composite. The frequency of repairable fractures was the highest in glass fiber post plus everX composite (91.66%) and the lowest in Filtek conventional post and core group (66.66%).

A systematic review and meta-analysis by Jurema et al. [14] showed that use of fiber post for restoration of endodontically treated teeth increased their FR. They attributed this improvement to better stress distribution in tooth structure. Stress is accumulated at the cervical part of the teeth that have lost a large portion of their structure due to extensive caries and endodontic treatment. Thus, fiber post placement aids in better stress distribution and higher FR [15]. Accordingly, Mojarad and Selahbarzin [8] compared the FR of severely damaged primary incisors restored with quartz-fiber post, prefabricated orthodontic wire posts, and composite resin posts. In line with the present findings, they found that fiber posts conferred higher FR than composite resin and y-shaped orthodontic wire posts in severely damaged primary incisors. In addition to the enhancement of FR, some studies reported that fiber posts improved the prognosis of teeth after fracture. They showed that teeth restored with fiber post are repairable in case of fracture; in other words, fracture occurs above their CEJ, or in the coronal third of the crowns, and does not extend to the roots [14, 16, 17]. It should be noted that the obtained fracture resistance for some specimens was higher than the compressive stress of enamel (380 MPa) or dentin (297 MPa) because, in the present study, not only the chemical adhesion of composite to enamel but also the intracanal mechanical adhesion provided by using a prefabricated glass post and composite post with 3 mm height were used for build-up of the teeth. Thus, scientifically, it was expected for the complex of tooth, post, and resin, to have a fracture resistance higher than that of enamel or dentin considering the thickness and mechanical properties of each component, compared with the fracture resistance of each component alone. Similarly, a previous study reported the fracture resistance of primary anterior teeth restored with a bulk-fill composite to be 480 N [9] and another study reported the fracture resistance of teeth restored with intracanal post and composite to be as high as 500 N [1, 18].

Different types of fiber posts are available in the market including carbon, quartz, and glass fiber posts. However, only glass fiber posts can increase FR, which may be due to the similarity of elastic modulus of glass fiber posts to that of dentin, which results in better stress distribution [19]. Uctasli et al. [20] evaluated the FR of maxillary incisors restored with different post and core and full-crown restorations fabricated from a direct conventional composite (PFC, G-aenial Anterior, GC, Tokyo, Japan) or indirect CAD/CAM composite (Cerasmart 270 and glass ceramic LiSi Block from GC). The teeth were restored with SFRC post and core, dual-cure composite post and core, SFRC fiber post and core, and dual-cure composite fiber post and core. Consistent with the present results, they showed that restorations with fiber post had higher FR under static loads. Their results were different from the findings of Garoushi et al. [11] and Bijelic et al. [12] who found no significant difference in FR of SFRC post and core and fiber post restorations. It should be noted that everX Flow SFRC composite was used in the present study and also by Uctasli et al. [20] while Garoushi et al. [11] and Bijelic et al. [12] used packable composite. In the present study, the use of prefabricated glass fiber posts along with Scotchbond Universal adhesive and RelyX Luting Plus cement created a homogenous mixture that enhanced the FR compared with no use of fiber post.

The everX Flow SFRC used in the present study had high fracture toughness and flexural strength. According to Uctasli et al. [20] there is no other direct composite resin with fracture toughness higher than 2.6 MPa·(m)^1/2^; thus, it appears that extensive restoration of teeth with fiber-reinforced composite resin can aid primary anterior tooth crowns to tolerate the applied loads [20]. Kadkhodaei et al. [18] measured the FR of prefabricated glass fiber posts with different composite cores used for restoration of primary anterior teeth. They reported that the FR was the highest in prefabricated glass fiber post plus conventional composite build-up. Their results were consistent with the present findings. In contrast, Abduljawad et al. [15] reported that the use of carbon fiber or glass fiber posts did not increase the FR of endodontically treated maxillary incisors with class III restorations. The lowest FR was reported in teeth restored with glass fiber post and the highest FR was reported in control group without fiber post in their study. This finding indicates that sound coronal structure plays a pivotal role in FR, and in teeth with class III restorations, further removal of tooth structure for post space preparation weakens the tooth. Thus, it may be concluded that residual dentin after endodontic treatment plays a pivotal role in FR of endodontically treated teeth [15]. In the present study, the selected teeth had cervical third of the crown, which were different from the teeth used by Abduljawad et al. [15].

In the present study, the FR of teeth restored with everX composite post and core was not significantly different from the FR of teeth restored with Filtek bulk-fill and Z250 conventional composite groups. Shafiei et al. [21] evaluated the restoration of endodontically treated teeth with conventional composite and bulk-fill composite reinforced with polyethylene fiber posts and demonstrated that fiber-reinforced composite resins had higher FR. They separately added polyethylene fibers to the buccal and lingual tooth surfaces. Also, brand of composite, type of tooth, cavity size, and many other confounders can affect the results. However, similar to the present study, they found no significant difference between bulk-fill and conventional composite resins. A clinical study by Solanki et al. [22] showed that none of the endodontically treated teeth restored with everX composite had undergone fracture at the 1-year follow-up. EverX composite resin is recommended for dentin replacement in high-stress-bearing areas especially in large cavities of vital and nonvital posterior teeth. It is composed of a resin matrix, quartz microfibers, and a nonorganic filler [6]. In everX composite, E-glass short fibers are randomly dispersed in a resin matrix containing barium silicate fillers. These fibers have 3 mm length and enhance the FR. SFRCs highly resemble dentin in terms of microstructure and mechanical properties and are recommended as a bulk base or build-up core in large cavities of vital and nonvital teeth [7]. They can tolerate masticatory forces and are recommended for posterior areas [23]. Patnana et al. [24] used glass-fiber-reinforced composite and Filtek Z250 conventional composite for conservative restoration of the incisal edge of maxillary incisors and found a significant difference in FR of the two groups. Differences between their results and the present findings can be due to differences in cavity preparation designs, the extent of cavities, and no conduction of thermocycling in their study since thermocycling decreases the FR [25]. Mosharrafian et al. [9] found no significant difference in FR of teeth restored with SonicFill bulk-fill composite post and core and Z250 composite post and core, which was similar to the present findings, suggesting that bulk-fill composite can be used instead of conventional composite in primary teeth to save time.

In vitro design was a limitation of this study which limits the generalizability of the results to the clinical setting. Future clinical trials are recommended on quality and FR of different restoration protocols in primary anterior teeth over long periods of time.

## 5. Conclusion

Within the limitations of this in vitro study, the results showed that restoration of severely damaged primary maxillary central incisors with glass fiber post and everX composite reinforced with short fibers enhanced their FR and increased the chance of reparability in case of restoration fracture. This technique may be recommended for the restoration of primary anterior teeth since it is simple and saves time.

## Figures and Tables

**Table 1:** FR of the five groups (*n* = 12) in Newtons (N).

	Mean	Standard deviation	Standard error	95% Confidence interval for mean		Minimum	Maximum
				Lower bound	Upper bound		
EverX + fiber post	482.68	80.80	23.32	431.34	534.02	374.69	595.17
Bulk + fiberpost	400.16	82.76	23.89	347.57	452.74	253.44	539.60
EverX	398.21	95.02	27.43	337.83	458.59	232.94	558.97
Bulk fill	357.87	76.76	22.16	309.10	406.65	226.37	495.65
Z250	316.93	57.31	16.54	280.52	353.35	245.84	416.93
Total	391.17	94.68	12.22	366.71	415.63	226.37	595.17

**Table 2:** FR of the five groups (*n* = 12) in megapascals (MPa).

Group	Mean	Standard deviation	Standard error	95% Confidence interval for mean		Minimum	Maximum
				Lower bound	Upper bound		
EverX + fiberpost	19.30	2.68	0.77	17.59	21.01	15.42	22.89
Bulk + fiber post	15.93	3.19	0.92	13.90	17.96	10.13	20.75
EverX	15.81	3.50	1.01	13.59	18.04	9.70	21.49
Bulk fill	14.34	3.01	0.86	12.42	16.25	9.43	19.82
Z250	12.71	2.28	0.65	11.26	14.16	9.83	16.67
Total	15.62	3.60	0.46	14.69	16.55	9.43	22.89

**Table 3:** Pairwise comparisons of the groups regarding FR.

Group (I)	Group (J)	Mean difference (I–J)	Standard error	Sig.	95% Confidence interval	
					Lower bound	Upper bound
EverX + fiber post	Bulk + fiber post	3.37	1.20	0.05	−0.04	6.78
	EverX	3.49^*∗*^	1.20	0.04	0.07	6.89
	Bulk	4.96^*∗*^	1.20	0.001	1.54	8.37
	Z250	6.58^*∗*^	1.20	0.000	3.17	9.99

Bulk + fiber post	EverX + fiber post	−3.36	1.20	0.05	−6.78	0.04
	EverX	0.12	1.20	1.00	−3.29	3.52
	Bulk	1.59	1.20	0.68	−1.82	5.00
	Z250	3.22	1.20	0.07	−0.19	6.62

EverX	EverX + fiber post	−3.48^*∗*^	1.20	0.04	−6.89	−0.07
	Bulk + fiber post	−0.11	1.20	1.00	−3.52	3.29
	Bulk	1.47	1.20	0.74	−1.93	4.88
	Z250	3.10	1.20	0.09	−0.31	6.51

Bulk	EverX + fiber post	−4.96^*∗*^	1.20	0.001	−8.37	−1.54
	Bulk + fiber post	−1.59	1.20	0.68	−5.00	1.82
	EverX	−1.47	1.20	0.74	−4.88	1.93
	Z250	1.62	1.20	0.66	−1.78	5.03

Z250	EverX + fiber post	−6.58^*∗*^	1.20	0.000	−9.99	−3.17
	Bulk + fiber post	−3.21	1.20	0.07	−6.62	0.19
	EverX	−3.10	1.20	0.09	−6.51	0.31
	Bulk	−1.62	1.20	0.66	−5.03	1.78

^*∗*^The mean difference is significant at the 0.05 level.

**Table 4:** Frequency of repairable and irreparable fractures in the five groups.

Fracture mode	EverX + fiber post	Bulk-fill + fiber post	EverX	Bulk fill	Z250
Irreparable	8.34	16.67	16.67	16.67	33.34
Reparable	91.66	83.33	83.33	83.33	66.66

## Data Availability

The data used to support the findings of this study were supplied by corresponding author under license and will be available on request. Requests for access to these data should be made to the corresponding author.
